# Development and evaluation of an electronic diary for recording patient reported outcomes in the overt study

**DOI:** 10.1186/1745-6215-16-S2-O64

**Published:** 2015-11-16

**Authors:** Duncan Appelbe, Oluseun Adeogun, Nicholas Webb, Rabiya Majeed, Malcom Lewis, Paula Williamson

**Affiliations:** 1 Clinical Trials Research Centre, The University of Liverpool, Liverpool, UK; 2 Department of Biostatistics, University of Liverpool, Liverpool, UK; 3 Royal Manchester Children’s Hospital, Central Manchester University Hospitals NHS Foundation Trust, Manchester, UK

## 

The OVERT study is a pilot randomised controlled trial to compare the effectiveness of intravesical onabotulin toxin A with extended release tolterodine in the management of children aged 7-16 with Idiopathic Overactive Bladder.

The primary and secondary outcomes for this study are recorded in a bladder diary, as recommended by the International Children's Continence Society. In order to collect these data a web based diary (C#/MVC .Net/MySql Database) has been developed to make it easier for study participants to record the data required by the study. Feasibility data suggested that the vast majority of potentially eligible children and families have access to the internet.

Participants included in the study are required to complete two diaries prior to randomisation into the trial, along with four symptom assessment questionnaires post randomisation. Each diary/questionnaire is completed at four time points during the day (morning, afternoon, evening and after going to bed) for seven days, with the questions presented to the participant varying based on the type of day (volume measuring or non-volume measuring) and the questionnaire type.

The eDiary is configured with different roles so as to facilitate data entry by the participant, entry of diaries completed on paper by a research nurse and a review of data by clinical staff, thereby ensuring that all data can be entered into the application.

This paper will discuss the development process undertaken to create the eDiary, present results from the evaluation of the system and future plans to enhance this application.

